# Defining the clinician’s role in early health technology assessment during medical device innovation – a systematic review

**DOI:** 10.1186/s12913-019-4305-9

**Published:** 2019-07-23

**Authors:** Vinayak Smith, Ritesh Warty, Amrish Nair, Sathya Krishnan, Joel Arun Sursas, Fabricio da Silva Costa, Beverley Vollenhoven, Euan Morrison Wallace

**Affiliations:** 1 Department of Obstetrics and Gynaecology, Monash University, 252 Clayton Road, Clayton, Victoria 3168 Australia; 2 Biorithm Pte Ltd, 81 Ayer Rajah Crescent 03-53, Singapore, 139967 Singapore; 3 Department of Paediatrics, Rockhampton Base Hospital, Canning Street, Rockhampton City, Queensland 4700 Australia; 4 Department of Gynecology and Obstetrics, Ribeirão Preto Medical School, Ribeirão Preto, São Paulo, Brazil

**Keywords:** Health technology assessment, Medical device, Innovation, Needs analysis, Efficacy, Safety, Clinician, Clinical strategy, Framework

## Abstract

**Background:**

Early Health Technology Assessment (EHTA) is an evolving field in health policy which aims to provide decision support and mitigate risk during early medical device innovation. The clinician is a key stakeholder in this process and their role has traditionally been confined to assessing device efficacy and safety alone. There is however, no data exploring their role in this process and how they can contribute towards it. This motivated us to carry out a systematic review to delineate the role of the clinician in EHTA as per the PRISMA guidelines.

**Methods:**

A systematic search of peer reviewed literature was undertaken across PUBMED, OVID Medline and Web of science up till June 2018. Studies that were suitable for inclusion focused on clinician input in health technology assessment or early medical device innovation**.** A qualitative approach was utilised to generate themes on how clinicians could contribute in general and specific areas of EHTA. Data was manually extracted by the authors and themes were agreed in consensus using a grounded theory framework. The specific stages included: All stages of EHTA, Basic research on mechanisms, Targeting for specific product, Proof of principle and Prototype and product development. Bias was assessed utilising the NICE Qualitative checklist.

**Results:**

A total of 33 articles met the inclusion criteria for the review. Areas identified in which the clinicians could contribute to EHTA included: i) needs driven problem solving, ii) conformity assessment of MDs, iii) economic evaluation of MDs and iv) addressing the conflicts in interest. For clinicians’ input across the various specific areas of EHTA, an innovation framework was generated based on the subthemes extracted.

**Conclusions:**

The following review has identified the various segments in which clinicians can contribute to EHTA to inform stakeholders and has also proposed an innovation framework.

**Electronic supplementary material:**

The online version of this article (10.1186/s12913-019-4305-9) contains supplementary material, which is available to authorized users.

## Background

### The medical device industry and early innovation

The medical device (MD) industry is one that is in the stage of accelerated growth globally. It currently has a compounded annual growth rate (CAGR) of 5.3% per annum and is projected to be worth 674.5 billion USD by 2022 [1]. The industry is predominantly made up of small and medium size enterprises [80%(SMEs)] which demonstrate a strong commitment towards research and development (R&D) and direct 8–12% of their revenue towards it [2]. This picture of R&D focus, however, should be interpreted with caution as most SMEs are scarce on resources, especially at early stages of their inception [3]. This scenario helps frame one of the main conundrums encountered in the industry. As a result of the pressure to succeed, most of these SMEs have their R&D pipelines motivated by factors which impact their survivability, rather than being able to divert their attention and understanding towards developing and implementing a framework towards successful device innovation [4–6]. These formative years in the MD innovation process, however, are the most critical to the device outcomes and have a huge bearing on its success. Importantly, it is only during this time at which assumptions can be deeply explored and design and development changes can be adopted to provide the most optimal outcome for the MD [7–9].

### Early health technology assessment (EHTA)

Taking this conundrum into consideration, several governmental organisations and researchers realised the necessity for decision support tools to be developed to provide innovation guidance for these SMEs during this time with high levels of uncertainty [7, 10, 11]. Moreover, it was recognised that other stakeholders in early MD innovation (EMDI) required mechanisms they could depend upon to help justify and guide the adequate allocation of scarce resources [12–14]. It was out of this need from which EHTA was born.

Early health technology assessment (EHTA) is a field in its infancy within health policy. Its foundations lie in the pharmaceutical industry as a derivation of health technology assessment (HTA). HTA however had several shortcomings in informing the EMDI process. Firstly, it was crafted with the aim of assessing a technology after it was developed [11, 15–17]. Furthermore, its preliminary objective was focused on economic evaluation in tandem with clinical data in a bid to foster early iteration and maximise efficiency in relation to R&D [18, 19]. Recognising these limitations, policy researchers concentrated their efforts towards sculpting a more informative method, EHTA, which could be adapted to the EMDI process [11, 19, 20].

As with HTA, initial studies describing EHTA positioned it as a manner of economic evaluation in the early stages of product development [21]. Since that time, the expanse of the field has been significantly broadened in order to capture the various segments of the innovation process and remains in a state of constant evolution [22, 23]. Though a uniform decision is yet to be agreed upon, the recent seminal review by *IJzerman* et al. aimed to capture the plethora of issues and research in the field of EHTA by proposing a uniform definition. They proposed that EHTA should be defined as “all methods used to inform industry and other stakeholders about the potential of new medical products in development, including methods to quantify and manage uncertainty” [19]. This definition, though recognising industry as an important participant, aimed to include all stakeholders in the assessment and decision-making process. In addition, they built on the available literature in the field to extend its reach from traditional economic analysis to encompass the realms of identifying the unmet needs accurately, assessing stakeholder preferences, managing technological risk and simulating clinical trials. Importantly, they also delineated the factors motivating the EHTA process which included R&D strategy, preclinical market assessment, clinical trial design, market access and pricing strategies [19, 24].

### Making the case for early HTA

The available evidence at present alarmingly suggests that decisions in the EMDI are taken without decision support and with motivations spanning from enthusiasm, a need to pioneer and responding to market competition [22, 25].

EHTA addresses this through a structured process which addresses multiple facets and stakeholders in the EMDI and therefore allows for risk mitigation [9, 26]. This approach invariably translates to business intelligence with manufacturers having access to valuable knowledge such as R&D process flow, device design features, ergonomic factors, user perspectives, reimbursement potential and cost effectiveness [4, 16]. Importantly, this information is garnered at a time in the EMDI process when it is affordable and technically feasible to pivot to address them adequately [16, 24]. At this juncture, it also worth highlighting that there is a considerable difference between focussing on EHTA in comparison to carrying out market research on the device. EHTA aims to support health policy decision making on a product and demonstrate from an early stage the innovativeness of a product across multiple domains whilst the latter serves to inform the manufacturers alone and is not as rigorous and comprehensive as a methodology.

In addition, modern day EMDI is facing a climate where R&D is becoming more complex and resource intensive, healthcare budgets are shrinking and consumer demands are increasing [19, 26, 27]. In light of this, there is an increasing need for mechanisms to aid in the rationalisation of resources so as to divert them towards more promising technologies. EHTA addresses this by providing an objective manner of identifying pursuits which would be beneficial to society from an early stage and diverting attention away from development cycles which are unlikely to do so [24, 28, 29].

Furthermore, there is significant disconnect between the supply and demand side logics of the EMDI process as well. The supply side logic places emphasis on innovation policy outcomes, which considers wealth generation as a key consequence, whilst the demand side logic has a focus on health policy outcomes instead. EHTA helps bridge this gap between both these spectrums with the aim of bringing equilibrium to the process [16].

### Medical device development process

To put EHTA in context, a brief understanding of medical device development is necessary. Medical device development is a multi-faceted process which takes a concept from ideation and translates it to a product which can be utilised by the end users. The process fosters interdisciplinary collaboration amongst various professionals (i.e. clinicians, engineers, business development executives) with the eventual goal of delivering a successful innovation outcome [9, 19, 20, 24, 30] .

**Fig. 1:**
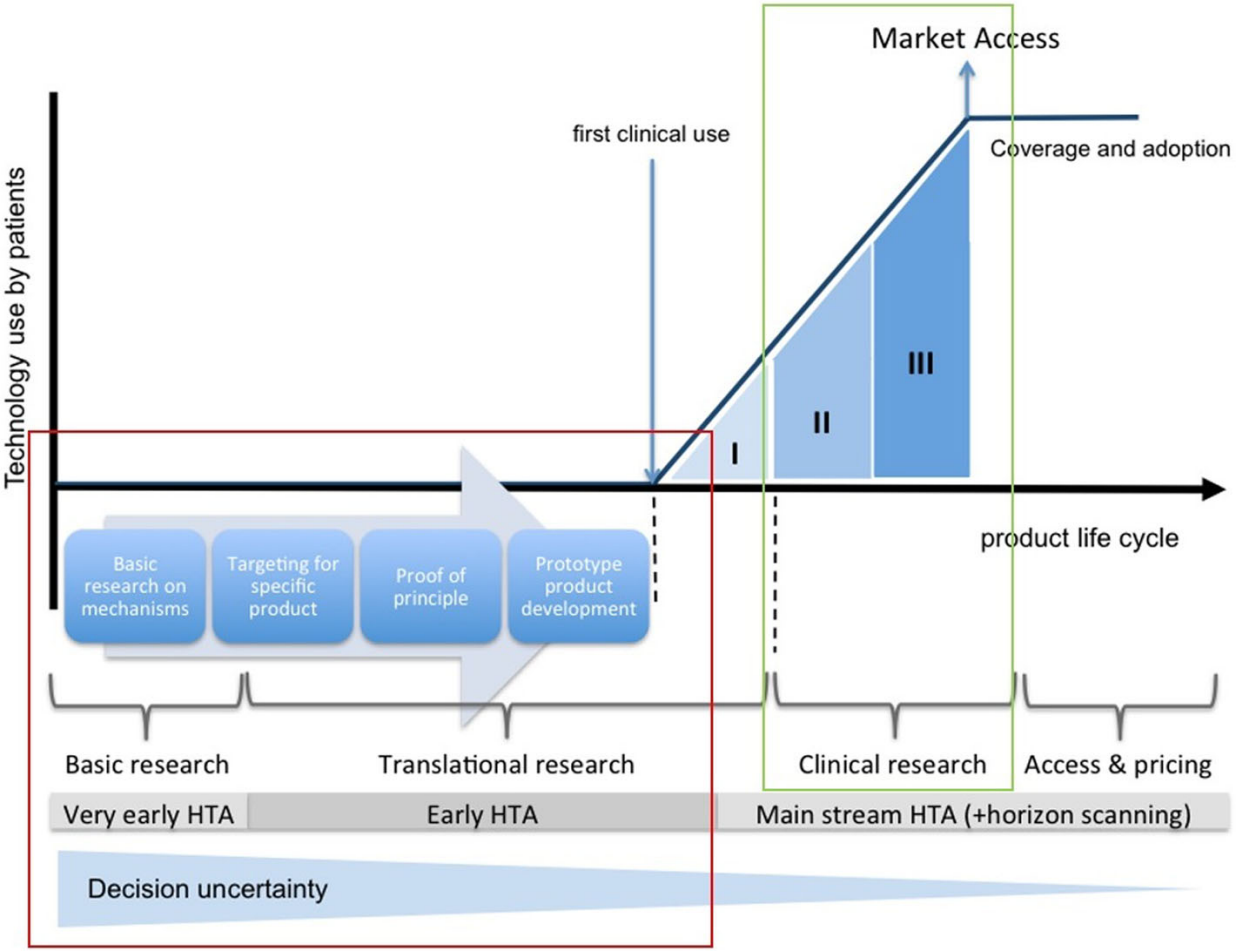
The medical device development process. The red outline illustrates the realm in which Early Health Technology Assessment (EHTA) occurs. The green outline illustrates the region where traditional clinical research influences the process. (Utilised with permission and adapted from *Ijzerman and Steuten*)

To date, several authors have described a variety of conceptual models to describe the various stages in the process and how they interact [12, 31–33]. Most of these models are presented in a linear fashion with separate decision gates. These ‘stages’ serve as areas in the cycle where stakeholders can iterate on product development on the basis of evidence being presented to them [4]. One of these models which has been utilised extensively in EHTA literature was proposed by *IJzerman and Steuten* as illustrated in Fig. 1 [24]. This allows for ease of visualization of the processes that allow for successful MD innovation. It should be reiterated however, that in reality, most of these processes are neither linear nor discrete in nature and tend to occur concurrently.

EHTA techniques are particularly of use during the periods of basic research and translational research (outlined in red). This is during a phase in the device life cycle where development is still ongoing and high-risk engineering and investment decisions are being made with a large level of uncertainty as to their future outcomes [9, 22, 24].

### And this matters to the clinician because?

A clinician is defined as ‘an individual who utilises a recognised scientific knowledge base and has the authority to direct the delivery of personal health services to a patient’ [34].

Traditionally, clinicians have been the main triggers for the development of novel MDs and their role is deeply intertwined with the EMDI process [33]. Their role on the EMDI however, has conventionally been restricted to conformity assessment, which involves assessing the effectiveness, comparative performance and safety features of the MD being developed (highlighted in green in Fig. 1) [24, 33].

The available evidence however, does suggest that the clinician does serve a function beyond conformity assessment alone. For instance, several authors have emphasised that in early translational work, the first and most important decision which guides the process of medical innovation is whether the product has a clinical need [24, 30, 33]. In addition, it is also known that MD developed with strong clinician input have greater clinical application and relevance as well [16].

Furthermore, it should also be highlighted that early clinician involvement can avert potential reverberations later in the EMDI as well. For instance, regulatory bodies have linked the paucity of robust, high quality studies in the regulatory approval process to the lack of clinical evidence generation early in the innovation process as well [10, 29, 35, 36].

Considering these findings, it would be safe to assume that the clinician is a key stakeholder in the EMDI process and can further contribute to the EHTA process.

### Objectives

Recognising the intimate relationship between the clinician and the EMDI, we sought to understand the areas in which they could contribute to EHTA. The area of interest is outlined in red in Fig. 1. As part of the multidisciplinary team (MDT) overseeing the process, clinicians are in a pole position to influence the EMDI and additional value can only be anticipated with clarity in how they can contribute to the EHTA process. Despite this, there appears to be no data in the literature to provide guidance on the role of the clinician as well as how they can effectively contribute to this EHTA process.

Identifying this gap in knowledge was a primary motivation for us to carry out a qualitative methods systematic review to delineate the role of the clinician in the EHTA process. The primary objective of this review is to provide clarity and a framework to researchers in the field of EHTA regarding areas and manners in which the clinician could influence EHTA.

## Methods

### Data sources

A search was carried out across 3 databases (PUBMED, OVID Medline and Web of science) by VS, RW and SK as per the PRISMA guidelines up till June 2018.

Theoretical and application studies were considered suitable for this systematic review. Application papers were defined as any studies which utilised empirical data in formulating their conclusions. All other papers were considered theoretical in nature. No exclusions were made for the year of publication. Articles of relevance identified from the reference lists of studies searched were suitable for inclusion in the review as well. Only articles in English were considered suitable for the following review.

### Search strategy

**Fig. 2:**
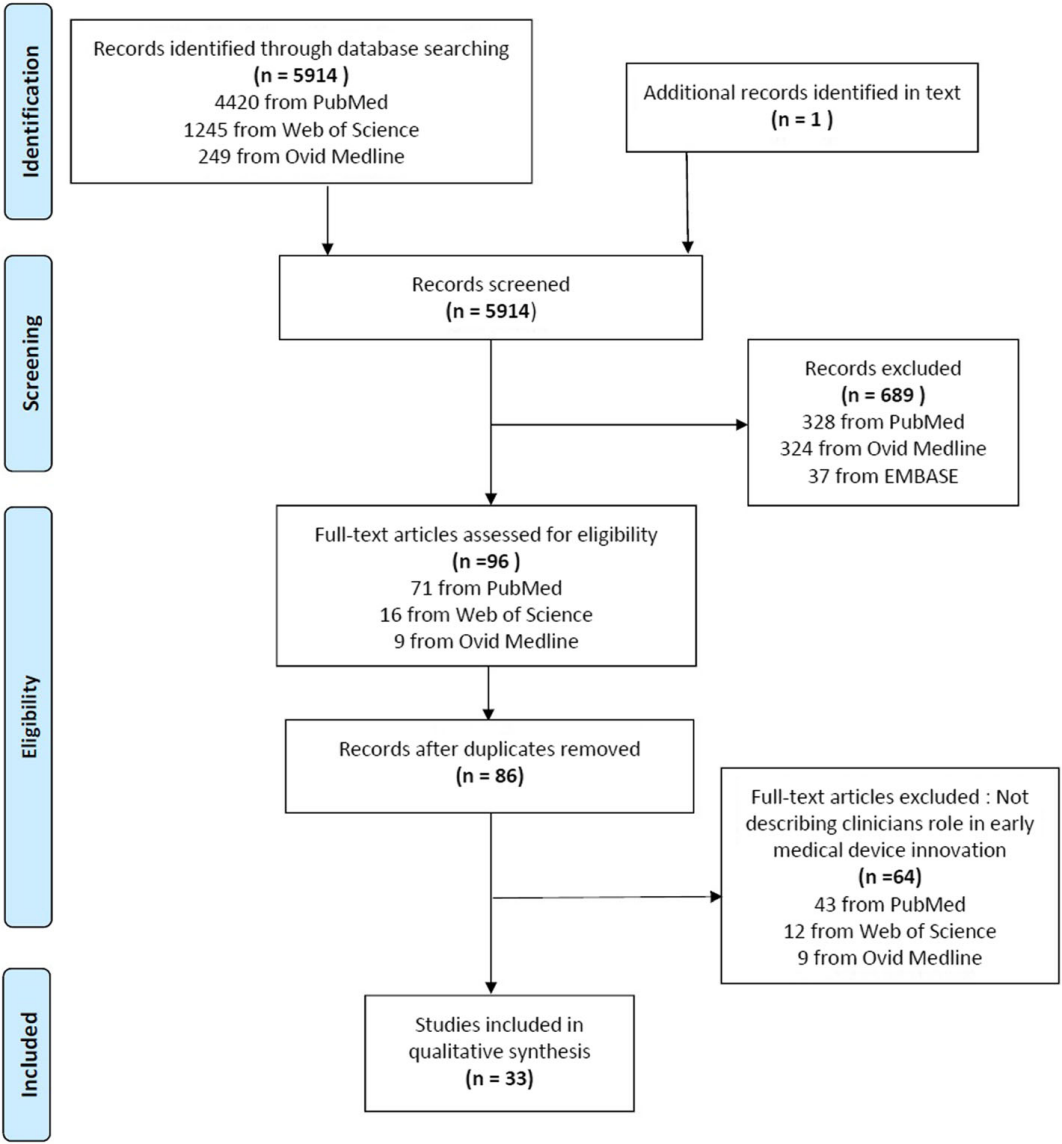
PRISMA flow chart for the study

The search strategy utilised is illustrated in Additional file 1. Search terms utilised include combinations of the following: medical device*, clinician*, health technology assessment and medical device development. Figure 2 depicts the flow of information through the various phases of the systematic review.

### Study selection

Based on the search strategy employed in Additional file 1, no articles were found describing the specific role of the clinician in EHTA. As such, the inclusion criteria for the review was any article focussing on the role of the clinician in health technology assessment or early device innovation. This was confined to articles focusing on device development prior to the commencement of phase I trials as suggested by *Markiewicz* et al. [22]*.* Hospital based HTA techniques and studies focusing on developing organisational HTA frameworks were not considered for inclusion as they occur late in the medical device innovation process (after Phase 1 trials), thereby limiting their relevance.

Citations were screened for suitability of inclusion in study and full text articles were ordered and read to determine short listing of the article for further review. Citations were screened by VS, RW, JAS and SK. Following shortlisting, the articles were read thoroughly by RW, VS, JAS and AN, and inclusion for the review was based on consensus.

### Data collection process

Given the nature of the topic and the lack of available evidence, a qualitative approach was employed for evidence synthesis.

A grounded theory framework was employed to allow examination of the clinician’s role in EHTA. This approach allows for development of theories and concepts grounded in the study data [37]. This was deemed suitable as the focus of grounded theory is on examining psycho- social processes of behaviour, in particular regarding how and why people behave in certain ways in similar contexts. Furthermore, it is one which is inductive in nature as well, allowing for the transition from specific to general concepts in explaining phenomena [38].

Segments where clinicians could influence aspects of EHTA were defined as per the stages highlighted in red on Fig. 1 – an approach similar to the review carried out by *Markiewicz* et al. [22]. These include a) basic research on mechanisms, b) targeting for specific product, c) proof of principle and d) prototype product development.

Articles shortlisted for inclusion were read thoroughly by VS, RW and AN in tandem. In line with the objectives of the study, the articles were examined for references to the clinician’s role in EMDI and the stage of EHTA (if available) it impacted upon. An initial thematic content analysis was then carried out by all three authors in tandem for each article in a systematic manner through immersion in the data, coding and subsequent data interpretation. This was to reduce researcher biases in summarising the content of the various sources of literature and increase credibility.

Reflexivity is a process which has been proposed during qualitative analysis procedures to aid in addressing self-bias, preferences and theoretical predispositions [39]. All researchers had anecdotal and experiential information regarding the roles played by clinicians in EHTA from their experience in medical device development initiatives they had been involved in. As such, during the analysis, a preliminary reading of the available literature was undertaken to inform the research question rather than to provide in depth understanding on the issue in order to limit the perpetuation of preformed hypotheses on themes.

Independent analyses were subsequently compared for commonalities and differences and a consensus summary for each article was compiled in Additional file 2.

### Data items

Data items of interest included study design, year of publication, article type (theoretical or application), primary issue raised in the article, impact of issue in EHTA, stage of EHTA which the issue had significant impact on and supplementary issues described by the article.

### Assessment of study quality

Assessment of study quality was carried out by utilising the NICE (UK) Qualitative Appraisal Checklist (Attached as an Additional file 3). This is presented in Table 1. The assessment was carried out by RW, VS and SK individually and consensus was reached in discussion before an overall score was given for the quality of the study.

**Table 1:** NICE qualitative appraisal checklist ratings for studies included in review

NICE Qualitative Assessment Tool	Number of studies	Percentage of all studies (%)	Studies
++	12	36.4	[11, 22, 29, 32, 40–47]
+	13	39.4	[27, 48–59]
–	8	24.2	[60–67]

++ − all or most of the checklist criteria have been fulfilled and the conclusions of the study are unlikely to be altered by those criteria that have not been fulfilled, + some of the checklist criteria have been fulfilled and where they have not, the conclusions of the study are unlikely to be altered, − few or none of the criteria checklist have been fulfilled and the conclusions are very likely to be altered

### Synthesis of results

Building on the initial thematic analysis carried out in 2.4 and documented in Additional file 2, a secondary thematic analysis was carried out to address the primary objectives of the systematic review (presented below). For this stage, studies were eligible for classification across multiple themes the summarised data was independently analysed by VS and RW and independent categories were identified.

Reflexivity was taken into consideration during the synthesis of results by using AN as a moderator for the themes generated in unison. He was involved in generating Additional file 2 and was thus aware of the general content of the included articles. He was also made to read seminal text of the clinicians role in early medical device innovation as well as EHTA so as to have a ‘content expert’ on the various roles of the clinician as well [19, 22, 32, 33, 46].

These analyses were subsequently compared for commonalities and differences and recurring themes and subthemes were developed with AN in an attempt to establish data which was broad as well as theoretically grounded as well. Eventually, all themes and subthemes were generated following consensus between VS, RW and AN.

For the analysis, the two areas of interest were:.

#### Areas in which clinicians can contribute to the EHTA process

This thematic area was focused on specific mechanisms through which the clinician can be engaged in EHTA and influence the EMDI process.

#### Clinicians role across the specific stages of EHTA

This thematic area focussed on the establishing a framework for the clinician to partake across the various phases of EHTA (all phases, basic research on mechanisms, targeting for specific product, proof of principle and prototype product development). The motivations behind this was to function as a guide for clinician integration at any phase of the EHTA process.

## Results

### Overview

For the following systematic review, 33 articles met the inclusion criteria. 24.2% (*n* = 8) papers were application papers and 75.8% were theoretical papers. The years of publication ranged between 1996 to 2017. Data which was extracted from each of the papers is tabulated in Additional file 2 which was utilised to generate the themes and subthemes.

### Primary objectives

**Fig. 3:**
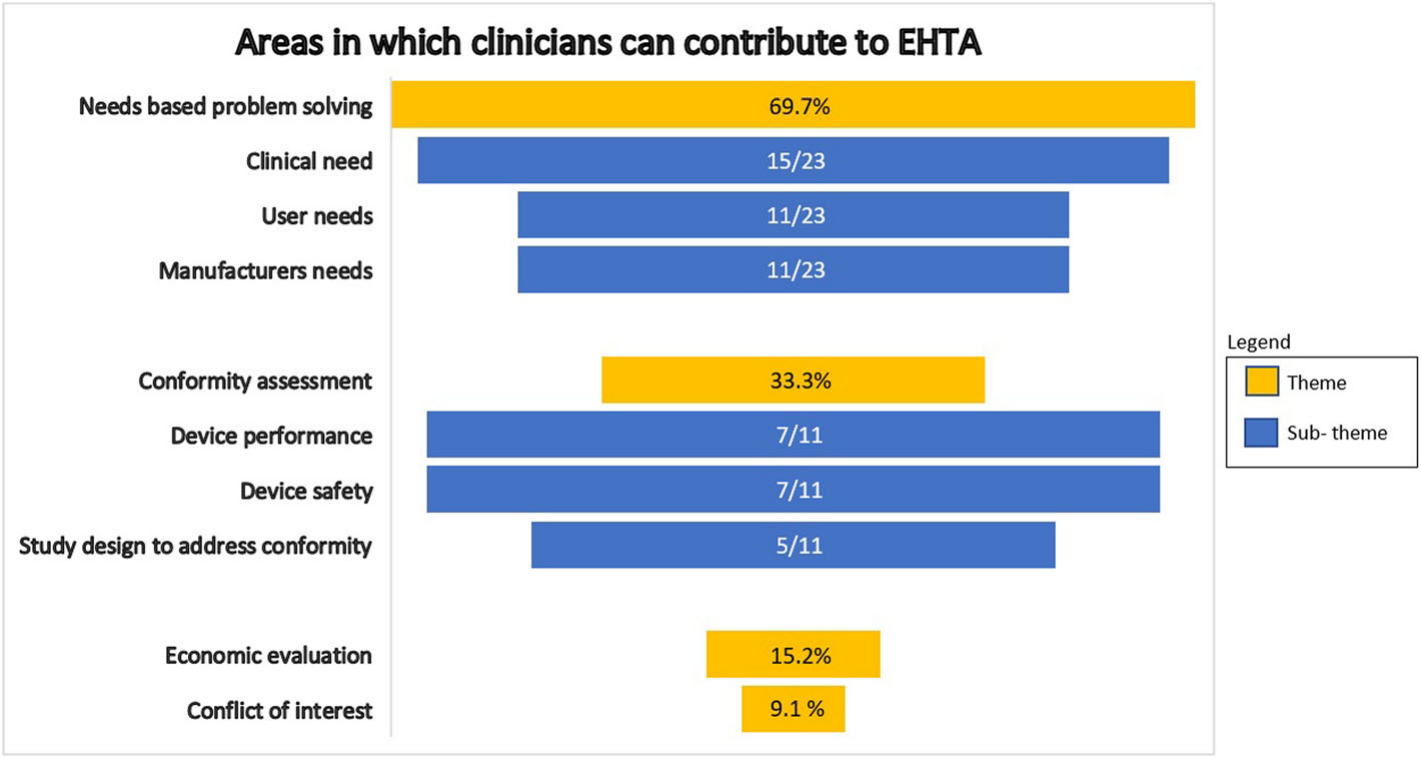
Areas in which clinicians can contribute to EHTA. Percentages in themes (yellow) are in comparison to all studies (*n* = 33) included in the review. Sub theme (blue) values are for all studies included in the theme

**Fig. 4:**
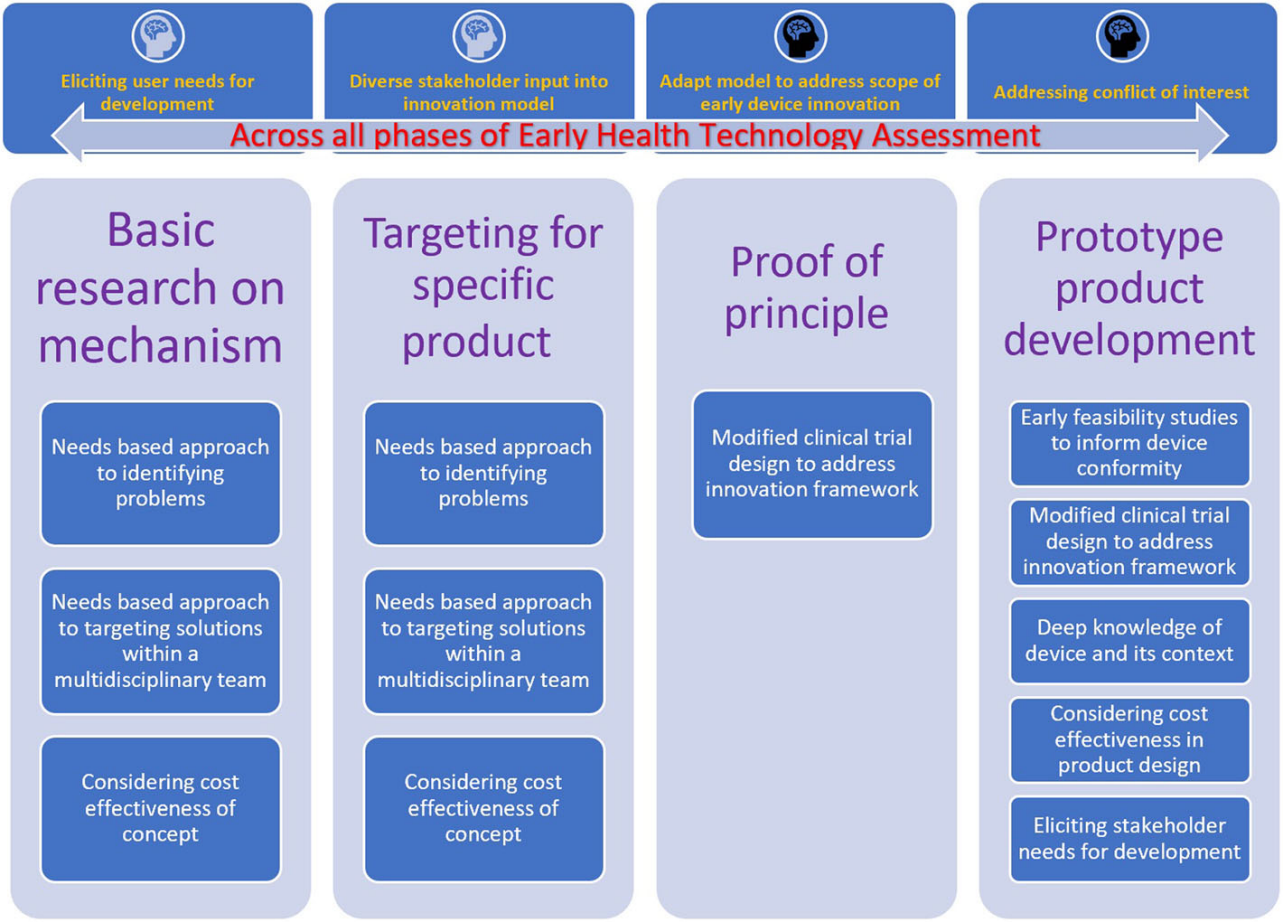
Framework through which clinicians can contribute to the various stages of EHTA

Table 2 contains a summary of the themes and subthemes to address the question of areas in which clinicians can contribute to the EHTA process and is graphically represented in Fig. 3. The means by which the clinician can contribute and interact in the EMDI process across the specific stages of EHTA is summarised in Table 3. Figure 4 further illustrates this as a framework to allow for ease of applicability of the findings.

**Table 2:**
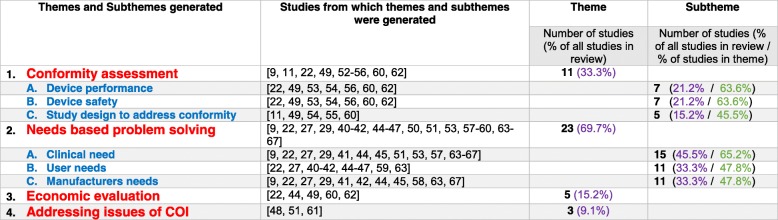
Areas in which clinicians can contribute towards the EHTA process. For the first column, themes are in *Red* and subthemes are in *Blue*. For the last column, *Purple font* denotes the proportion of studies highlighting the theme against all studies identified in the review. *Green font* denotes the proportion of studies highlighting the subtheme against the studies identified in the theme

**Table 3:**
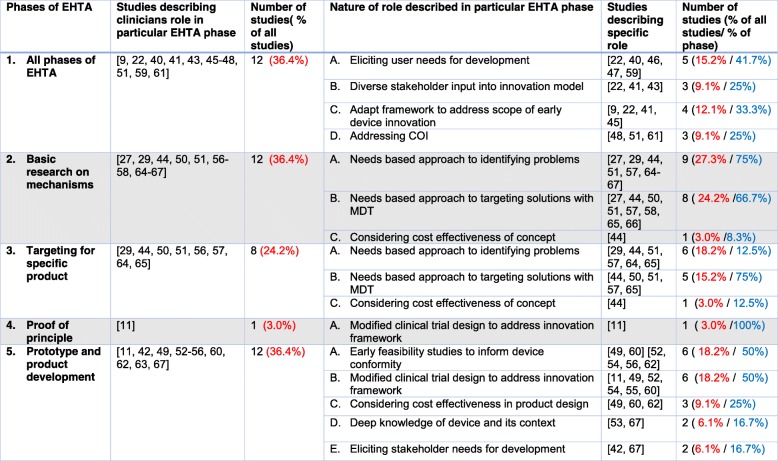
Framework through which clinicians can contribute to various stages of EHTA. COI- Conflict of interest, MDT- Multi disciplinary team. *Red font* denotes the proportion of studies highlighting the theme against all studies identified in the review. *Blue font* denotes the proportion of studies highlighting the nature of the role against the studies identified in that specific phase of Early Health Technology Assessment

As a whole, the themes and subthemes generated encompassed:

#### Needs driven problem solving

As a theme, the majority of studies (69.7%) described **‘needs driven problem solving’** as a key area of contribution by clinicians towards the EHTA process. The concept was also noted to have utility across all stages of the EHTA process with 15–27% of studies outlining its utility in some manner.

In terms of subthemes, assessing the **‘clinical need’** was given the highest level of importance with 45.5% of studies identifying it as an area of importance. In particular, the importance of this input seemed to be more impactful in the early stages of **‘basic research on mechanisms’** and **‘targeting for a specific product’** with between 15 and 27% of studies identifying it as **‘part of a needs based approach to identifying problems’** and **‘needs based approach to targeting solutions with a MDT’** .

In addition, 33.3% of studies in the review further highlighted the assessment of **‘user needs’** a sub theme of importance. Particularly 15.2% of the studies mentioned it to be of relevance across all stages of EHTA.

Finally, 33.3% of studies highlighted the assessment of **‘manufacturer needs’** as a subtheme of relevance.

#### Conformity assessment

33.3% of studies in this review identified **‘conformity assessment’** as a major theme with 18.2% of them suggesting a preponderance for it to be utilised in the **‘prototype and product development phase’**.

The subthemes generated highlighted the issue of **clinical performance**, **safety** and **study design** in particular.

#### Economic evaluation

15.2% of studies in the review identified **‘economic evaluation’** as a major theme. In addition, 9.1% have identified its area of importance to the stage of ‘**prototype and product development’**.

#### Addressing conflict of interest (COI)

9.1% of studies mentioned ‘**addressing COI’** to be a major theme and a theme across all phases of EHTA.

## Discussion

The discussion will aim to expand on the findings of the review and attempts will be made to highlight specific methods through which the clinician can partake in the process as well.

### Needs driven problem solving

#### Clinical need

The assessment of the **clinical need** is arguably one of the most important roles which the clinician must fulfil which impacts on all other aspects of EHTA.

The MD industry has historically been one which attempts to utilise technologies from other sectors to solve clinical problems. As such, the starting point often begins with a technological focus with hopes of translation to a device which solves a meaningful clinical issue [52]. This inevitably results in a form of confirmation bias where both manufacturers and clinicians alike look for a gap to fill with the technology which is neither clinically impactful nor a good use of resources [68].

Given the privileged position and immersion they have in patient care, the clinician is in a unique position to understand the pain points and subsequently demarcate these accurately. In addition, they function as a valuable resource in the MDT by providing insight into the feasibility of a solution given their knowledge of its context in the cycle of care of the patient. This lays the foundations towards developing a technology which can holistically address these areas of concern in contrast to moulding a use case on the basis of the technology alone [66–68].

Multiple resources are available to clinicians to establishing this clinical need and develop a statement of clinical need (SCN) to adequately define the scope of the clinical problem to be solved. Commonly utilised mechanisms include in depth reviews of the literature addressing the area of need, direct observations and interviews with key opinion leaders in the field [53, 66, 69]. Furthermore, as identified by this review, there is value in early interaction with professional societies as well. These societies could play a vital role in the needs assessment, given their expert panel, and aid in better defining the clinical gap. Importantly, they could also aid in identifying alternative use cases for the MD and provide introspection into how the MD may eventually fit into their current guidelines and eventually affect clinical management [70].

An in-depth discussion into the methods and areas of focus of the needs assessment is beyond the scope of this article. A thorough understanding of the factors to consider at this juncture can be sourced from the Stanford Biodesign model which articulates a clear approach to this process through their ‘Identification phase’ [33].

#### User needs

The ability of users to contribute to the various phases of the EMDI has been well established in the literature and is known to ascribe value to the process [46]. In particular, the earlier their involvement in the process, the greater the savings in terms of time and cost savings as earlier modifications can be addressed and instituted promptly [71].

It must be borne in mind however, that users encompass a wide range of individuals spanning from clinicians to end users (i.e patients and their family members). Understanding their individual needs is imperative for a successful device development process, product quality and safety [46, 59]. Furthermore, this contributes back towards the regulatory requirements for the specific device as well [46, 47].

Particular reference has to be made to the work of *Shah* et al. in their attempt to demarcate a framework through which user input can be elicited. These serve as excellent reference material for clinicians attempting to delineate user needs and integrate them into the device innovation process [46, 47, 59].

In the EHTA process, clinicians can employ a variety of quantitative methods to assess this including interviews and surveys through study design from early stages in the design process. It must be mentioned however, that in assessing user needs, there is substantial bias towards considering the needs of clinicians in contrast to end users [59]. It is vital therefore that a range of users are consulted to get a wide array of input as possible [72].

#### Manufacturers needs

Clinicians also need to have a thorough understanding of the value chain of the manufacturer involved in EMDI to generate the greatest impact during EHTA (i.e manufacturers business model,R&D process, regulatory strategy,go to market strategy) [24, 30].

A key advantage of this knowledge is the awareness it generates towards obtaining critical data which contributes towards this value chain during the EMDI process (i.e validating the value proposition of the MD, marketing information, forecasting adoption, price sensitivity) [46].

### Role of conformity assessment

‘**Conformity assessment’**, which entails characterising the clinical performance and safety of a device, is regarded as the core contributory area in EHTA for the clinician.

Historically, clinicians have been well-versed with conformity assessment by leveraging on their knowledge in specific areas of interest. The importance of demonstrating conformity assessment cannot be understated, as the MDs clinical utility, value proposition and the subsequent regulatory process hinge on it [9, 20, 27, 73].

#### Clinical performance

In demonstrating performance, clinicians should be aware that the burden of proof is heavily dependent on the ability to demonstrate that the device can perform its purported claims adequately (and equally to a comparator if available) to the regulatory bodies [74, 75].

It must be mentioned however that displaying clinical performance is a very separate concept to demonstrating **clinical effectiveness**. Establishing effectiveness sets the bar higher for manufacturers in terms of having to demonstrate that the MD performs better than its available comparators [74, 75]. In the context of modern-day healthcare innovation, this ties in closely with generating “better outcomes at lower cost”. As such, establishing effectiveness ties in favourably with future late HTA and reimbursement decisions. It is also expected that demonstrating effectiveness will eventually become a requirement of the regulatory approval process as evidenced by the New European Medical Device Regulation. As such, from a strategic point of view, this is a concept that the clinician should grasp early in a bid to increase their value add to the process in general through clinical trial design even from the stage of early feasibility studies.

In establishing these endpoints, *Hamilton* offers valuable insight into the benefits of a thorough literature review prior to the commencement of the entire collaboration process for identification of useful outcome measures which can be utilised to characterise device performance [53]. The due diligence at this point will also entails an in depth understanding of the device and previous use cases through the Investigator’s Brochure (IB) provided by the manufacturer.

When discussing the evidentiary burden for medical devices, the risk profile of the device needs to be taken into consideration as well. For the various regulatory pathways, the level of evidence required for the devices varies significantly and this should be taken into consideration from the beginning in designing the feasibility studies. Essentially, this allows for extrapolation of the data from these studies to guide the design and pre-empt the logistical issues which may affect the pivotal trails for the device [76]. The interplay of these factors are captured in the EHTA framework (Fig. 4), as identified by 6.1% of studies, through **‘deep knowledge of the device and its context’**.

#### Safety

For the clinician, there is also a necessity to prove through conformity assessment that the MD is safe for patients and does not pose any threat to their health.

The relevant information for these end points can be sourced from the IB provided by the manufacturer. This describes any prior adverse reactions encountered through device use and encompasses a risk management tool which outlines potential safety risks of the device to the patient and how to manage those risks as well. Further device specific risks can also be elicited from systematic review of the literature and device registries of similar or predicate devices [22, 29].

#### Study design issues

One of the findings of this study is in relation to considerations towards study design during the EHTA process. This is to be expected given the broad group of contrivances which are considered to be MDs which invariably impacts on conformity assessment. 15.2% of studies in this review identified **‘study design to address device conformity’** as a subtheme. This issue was further elaborated on in the framework generated too. The review identified the need for **‘modified clinical trials to address the innovation framework’** (3–18.2%), which highlighted the need for modified and adaptive trial designs to cope with the device specific peculiarities in assessing MDs.

There are a number of factors specific to MDs which enact this influence on study design. Firstly, MDs undergo cycles of rapid of change, between 18 and 24 months in duration, during their development. Subsequent iterations of the device often include updates of incremental innovation. As such, the right time to assess the device is often a subject of debate and confusion. In addition, it further raises questions of the generalisability of the MD being tested and the final product as well [52, 55, 74, 76, 77]. Also, there is a learning curve associated with its use, from both an individual and institutional perspective which can affect and compound its assessment [10, 55, 76, 78]. Furthermore, the studies may have significant confounding from the user profiles of the participants as well as the adjuvant therapeutics, if any, that these patients are utilising as [11]. Finally, the inability to blind for certain devices, such as implantables, is also a factor of note [10].

In the context of these limitations, a move to address them must be propagated from angle of trial design itself. Industry generally has propagated the notion that the randomised controlled trial (RCT) is the design of choice to assess MDs. As can be discerned from the characteristics of MDs, the RCT for EHTA may not be the gold standard as it will provide only limited information as feedback into the early innovation process. With certain high-risk devices, the RCT itself may not be a feasible option and be unnecessary or inappropriate [74, 75]. As such, adaptive trial designs, incorporating Bayesian models are increasingly being utilised to address these issues in comparison to the frequentist design. These trial design methods are beyond the scope of this review but can be discerned from the following studies [79–81].

### Economic evaluation

The available evidence suggests that MD uptake has driven healthcare costs up globally [82–84]. This paints a worrying picture when placed in context of the modern-day healthcare system where medical reimbursement is continually decreasing. This provides the context for economic evaluation in EHTA which attaches importance to demonstrating better health outcomes and value to innovation for each dollar spent [82].

Traditionally, the responsibility of assessing value was left to physicians which understandably provided a myopic view of it [52]. This process has needfully evolved over time to include various stakeholders involved in the innovation process. The concept of value however, is diverse and the various stakeholders view value in a different light [85]. As such, finding middle ground and aligning these multiple perspectives has brought considerable challenge to the process itself.

Furthermore, this evolution also seems to have detached the clinician from the evaluative process itself [52]. One misconception which is increasingly being mooted by clinicians to justify this is with regards to the lack of real world application of cost utility analyses in their daily practice (i.e a clinician does not attach a cost to a blood pressure reading as it does not come with a bill). In addition, some clinicians attach considerable stigma to evaluating healthcare outcomes in terms economic metrics due to their beliefs that a cost cannot be attached to the sanctity of life or health which limits their participation in the process as well [86].

Thankfully, to date, the focus and development of EHTA has been centred on economic evaluation. The literature describes techniques such as headroom analysis and return on investment (ROI), which can be easily incorporated into the EHTA process by clinicians. These methods however, are beyond the scope of the current article but the following articles provide valuable insight into the techniques and their relevance [9, 19, 24, 30] .

### Addressing conflict of interest

The propensity for conflicts of interest (COI) to occur during participation in the EHTA process is one which is of significant concern to the clinician. 9.1% of studies mentioned ‘**Addressing COI’** to be a major theme and a theme across all phases of EHTA.

COI is a necessary evil during the EHTA process and in collaborations between clinicians and industry. As demonstrated by the review, the role of the clinician in EHTA is one which is vital and as such, a move to completely remove the clinician from enterprise is one which is unfounded and unrealistic [87, 88]. In recognising these shortcomings, there has been an increasing move towards managing these conflicts which can be sourced through Additional file 2 and the following studies [48, 51, 61].

### Framework through which clinicians can contribute towards EHTA

Both Table 3 and Fig. 4 provide an overview of the framework which clinicians can utilise to contribute to the EHTA process. Although not exhaustive in nature, it provides direction of key issues for consideration.

Of note is 33.3% of studies identifying **‘Adapt model to address the scope of early device innovation’**. This theme illustrates the variable nature of the EHTA process in relation to the MD being innovated upon. Clinicians should be aware that the EHTA process is one which is in a constant state of flux and, is not one that is linear and stagnant. This theme highlights the need for clinicians to remain vigilant to these MD specific peculiarities, such as the risk profile of the device, and adapt the EHTA process as appropriate to address these.

## Limitations

One of the main limitations of this study is in the search strategy utilised. Although in line with the PRISMA guidelines, relevant information from grey document sources were not included in the search which may have provided a more holistic perspective of the clinician’s role in the process. These issues can be averted with the utilisation of information specialists in the generation of a search strategy in future work on the issue.

The quality assessment using the NICE quality assessment tool was aimed to provide an overview of the quality for the studies included in this review. As can be garnered, nearly 24.2% were of low quality. This was mostly in relation to the opinion pieces and commentaries which were examined for inclusion. Given the nature of the topic however, we deemed it vital to get as much perspective on the early medical device innovation process to generate the aforementioned data. In addition, this is an issue traversed often in qualitative research. Furthermore, these studies remain valuable in generating the themes identified in our review.

In addition, investigator bias and reporting bias in generating themes is an aspect that needs consideration as well. Attempts were made to address this by recruiting an MDT, comprising of clinicians and biomedical engineers involved in the bio design process, for this review to generate themes and subthemes. It must be borne in mind however, that certain elements of the area of interest may be substantially over or under represented as a result.

Furthermore, the proportions generated for themes and subthemes in 3.2 and 3.3 be depended upon to classify the importance of issues hierarchically given the diversity of topics and the manners in which they have been generated. This is intended to provide an overview and justification for the selection of the themes.

## Future research direction

One aspect of research direction that will benefit clinicians is the development of a specific framework for clinical trial design and adaptation for EHTA. Although our framework in Fig. 4 provides an overview of issues which can be addressed, there will be value in demarcating the exact factors which can be incorporated in trial design and how they feed back and add value to the EHTA process. This is one of the core focuses of future research of our group.

It should also be highlighted that previously, device innovation had a strong focus on ‘supply side’ factors in delineating lower cost, better efficacy and safety. Recently however, there has also been more interest in ‘demand side’ technologies which are more patient centric (i.e. non-invasive, telemonitoring) and less focussed on those historical tenets [52]. Future research can also be directed to delineate these supply side factors which could potentially affect the EHTA process in the future.

Finally, another element that is also worth evaluating would be the cost effectiveness attached to the process of EHTA as a process itself. Given the large investment in time, resources and capital directed towards it, the value of the process itself is yet to be evaluated.

## Conclusion

Involving the clinician will be an additional if not missing piece to the complex puzzle which is EHTA. As demonstrated by the findings of this review, areas in which clinicians can contribute include conformity assessment, needs based problem solving, economic evaluation and addressing issues of COI. In addition, this can take place across the various specific stages of EHTA as illustrated by the framework in Fig. 4**.** When carried out in a structured manner, one would anticipate that the feedback loop will allow for teams to pivot at a stage when it is technically feasible and affordable then at later phases where minimal change can be exerted over the technology or its applications. Furthermore, such an approach will aid in stakeholder making decisions on MD ventures in an informed manner as they are empowered with the knowledge to do so.

## Additional files

Additional file 1: Search strategy (DOCX 14 kb)

Additional file 2: Data extracted from studies to delineate areas in which clinicians can contribute to the EHTA process. (DOCX 46 kb)

Additional file 3: NICE qualitative appraisal checklist. (PDF 1073 kb)

## Data Availability

All data generated or analysed during the study is included in this published article and in the supplementary data.

## Abbreviations

COI: Conflict of information
EHTA: Early Health Technology Assessment
EMDI: Early Medical Device Innovation
HTA: Health Technology Assessment
MD: Medical device
MDT: Multi Disciplinary Team
R&D: Research and Development
SME: Small and Medium sized Enterprises
