# Supplementary material for: Defining the clinician’s role in early health technology assessment during medical device innovation – a systematic review
Source: BMC Health Serv Res. 2019 Jul 23;19:514. doi: 10.1186/s12913-019-4305-9 (PMC6651962; doi:10.1186/s12913-019-4305-9)
Supplement: Supplementary file 1 — Search strategy (DOCX 14 kb) [file 12913_2019_4305_MOESM1_ESM.docx]

**Search strategy**

For all searches no limits were placed on language or year of publication

1. **Database: PubMed**

Date of search : 24/06/2018

Search terms :

( medical device* innovation AND clinician* ) OR (health technology assessment AND medical device*) OR ( health technology assessment AND clinician*) OR (medical device development AND clinician*)

Results :

4420 citations identified and screened .

**71 articles** identified from citation screening and read through as full text. 28 articles included in review

1. **Database: Web of Science**

Date of search : 27/06/2018

Search terms:

( medical device* innovation AND clinician* ) OR (health technology assessment AND medical device*) OR ( health technology assessment AND clinician*) OR (medical device development AND clinician*)

Results:

1245 citations identified and screened. 16 articles identified from citation screening. 9 articles selected which overlap with previously identified articles.

**7 articles** read through as full text. 4 included in review.

1. **Database: Ovid Medline**

Date of search : 27/06/2018

Search terms:

( medical device* innovation AND clinician* ) OR (health technology assessment AND medical device*) OR ( health technology assessment AND clinician*) OR (medical device development AND clinician*)

Results

279 citations identified. 9 articles identified from citation screening. 1 articles selected which overlap with previously identified articles.

**8 article** read through as full text. 1 article identified in text for inclusion in review.
