# Supplementary material for: Defining the clinician’s role in early health technology assessment during medical device innovation – a systematic review
Source: BMC Health Serv Res. 2019 Jul 23;19:514. doi: 10.1186/s12913-019-4305-9 (PMC6651962; doi:10.1186/s12913-019-4305-9)
Supplement: Supplementary file 2 — Data extracted from studies to delineate areas in which clinicians can contribute to the EHTA process. (DOCX 46 kb) [file 12913_2019_4305_MOESM2_ESM.docx]

| **Number** | **Paper/ Year** | **Study type** | **Issues raised** | **Stage of EHTA process it affects** | **Impact of issue on EHTA** | **Supplementary information** |
| --- | --- | --- | --- | --- | --- | --- |
| **1** | **Abel et al, 1996** | Theoretical | Delineating the role of feasibility studies in early device innovation | Product and prototype development | Feasibility studies can be utilised to:   1. Test device efficacy 2. Device safety 3. Monitor for side effects 4. Document need for new technology 5. Estimate of cost effectiveness | Objective of feasibility studies are to:   - Prove that device does not do harm in comparison to standard of care - Prove if device can be used reliably on humans |
| **2** | **Baerlocher et al, 2009** | Theoretical | Conflict of interest | All phases | Physician industry collaboration is at high risk of COI. This may cause:   1. Loss of objectivity in patient care 2. Academic misconduct 3. Erosion in the trust placed by the public in scientists | Methods to address:   - Formulate strategies to actively address COI   - Current declarations of COI do not have any repercussions once they are declared - Formulate scopes of practice for clinicians to engage with industry   The collaboration as a whole cannot be curtailed as it will blunt the innovation process |
| **3** | **Baim et al , 2007** | Theoretical | Conflict of interest | All phases | Early stage companies pay physicians or provide equity stakes for remuneration. May cause:   1. Conflict between clinical role and commercial interests 2. Impact on trust with patient in terms of participation and recommendation of treatment   Questions to be raised in respect to research methods and validity of findings | Methods to address:   - Disclosure – informing all interested parties about COI - Escape – disengage from all activities with industry or from specific aspects i.e pivotal trials   Management- Senior non-conflicted clinicians can supervise and oversee the research |
| **4** | **Bouchard et al. , 1996** | Theoretical | Clinicians facilitate outcomes research through early feasibility studies- assesses the results of care provided to patient | Product and prototype development | Primary focus   1. Technical feasibility of study and safety of procedure in comparison to standards of care   Secondary focus   1. Cost effectiveness to assess impact on society 2. Development of monitoring programs to inform impact of technology | - Early assessment needs to consider the learning curve associated with the technique and factor it into study design - Outcomes for studies need to be clinically relevant when designed |
| **5** | **Bridgelal Ram et al , 2008** | Theoretical | Clinicians can facilitate a needs driven model to facilitate device development through **user engagement** | All phases of EHTA | User engagement can result in :   1. Faster time to market 2. Lesser device modifications 3. Devices that are easily accepted by the market and culturally appropriate 4. Design solutions which meet user expectations | - Low risk devices tend to have higher levels of user engagement in development in - Clinicians act as the knowledge transfer vessels between users and developers |
| **6** | **Caparelli et al , 2015** | Application | Clinicians can utilise feasibility studies to carry out EHTA | Product and prototype development | Areas of focus for EHTA include ( in order of importance) :   1. Device safety 2. Clinical effectiveness 3. Economic assessment 4. Social impact |  |
| **7** | **Castner et al , 2016** | Theoretical | Defining methods through which nurses can contribute to early device innovation | All phases of EHTA | Nurses can contribute to early device innovation through:   1. The needs assessment stage to identify a clinical practice gap 2. Planned brainstorm stage 3. Feasibility determination   Concept design and prototyping | - Methods utilised for assessing needs analysis included registry surveys, systematic reviews of the topic of interest, interviews and focus groups - Planned brainstorms stage is for idea generation and solution generation and includes eliciting all stakeholders   Feasibility determination assesses the feasibility of the idea considering device risk, resource requirements and operation risks associated with further development of the idea |
| **8** | **Conway et al, 2012** | Theoretical | Feasibility studies can be utilised to carry out EHTA | Product and prototype development | Areas of focus for EHTA of the device included:   1. Device design and ergonomics (length of IPC sleeve and degree of fit) to aid manufacturer 2. User acceptance of the device 3. Clinician feedback on device form and performance 4. Device safety | - Device design was incremental during the trial through user input i.e sleeve movement was noted as an issue early on and design modifications were made - Clinician and user feedback actively guided product development and selection of product to market - A learning curve for the device was identified during the trial and was identified as a potential confounder regarding the results |
| **9** | **De Ana et al, 2013** | Theoretical | Utilising stakeholder input to influence the innovation process | Product and prototype development | Stakeholder analysis can be balanced by focussing on the :   1. Voice of the customer (VOC) 2. Voice of the business (VOB) 3. Voice of the technology (VOT)   Patients can be interviewed independently or in focus groups to derive information on device utility and experience  Physicians can contribute particularly in listing factors that influence device use , reasons for choosing one device over another and additional features which would add value to them | - VOC incorporates views from the patients and payers – similar to traditional marketing - VOB incorporates views of the business in terms of strategic direction and influenced by regulation, economic and legal constraints - VOT incorporates views of the R&D team and their limitations in product design |
| **10** | **Demers - Payette et al, 2016** | Theoretical | Defining how a responsible research framework can contribute to early device innovation | Basic research on mechanism | Utilising a responsible research framework through:   1. Anticipation i.e Needs identification 2. Reflexivity 3. Inclusion i.e user involvement in innovation process   Responsiveness i.e feedback loops of views and knowledge into R&D | - Needs identification should focus on clinical, organisational , ethical and social impacts . It should attempt to identify evolving needs and adapt appropriately. Thought should also be given to future impact on the system as well |
| **11** | **De Passe, J , 2014** | Theoretical | Working within a multidisciplinary context in early device innovation. | Basic research on mechanisms and targeting for specific product | Multidisciplinary teams will allow for:   1. Needs based approach to early device innovation 2. Rapid pivoting based on stakeholder input in early innovation 3. Addressing pain points early in device innovation | - Stakeholders identified in the paper include clinicians, engineers, end users and investors as well |
| **12** | **Donovan and Kaplan, 2012** | Theoretical | 1. Role of clinician in early device development 2. Conflict of interest | Basic research on mechanisms and targeting for specific product  All phases of EHTA | Clinicians can help by:   1. Identifying unmet clinical need 2. Product development 3. Assessment of impact of technology on condition   Individual vs organisational conflict usually encountered in medical device development. Fostered by cash or equity stakes given as remuneration. May cause:  1. Loss of credibility of clinician  2. Loss of public support for project | - Clinicians play an integral part in study protocol development based on their understanding of device specifics and relevant end points in the clinical care pathway   Methods to address include:   - Protocol development:   Clinician should not be PI on pivotal studies   - Patient recruitment:   Clinician with COI should not be the only one tasked with recruitment and obtaining informed consent   - Performing the procedure   Clinician should not perform procedure and should only serve as a technical assistant   - Data entry - Clinicians with COI should not be tasked with direct data entry |
| **13** | **Geljins, A et al , 2013** | Application | Role of clinician in assessing value in early device development | Product and prototype development | Challenges in assessing a novel device:   1. Comparison arm is usually a very different treatment modality 2. Blinding is not feasible 3. Incremental nature of device during trials 4. Significant learning curve attached to device utility 5. Small trial sizes | - During the innovation process, new indications for use may be uncovered - Device utilisation tends to become better with time due to incremental innovation and learning curve – assessing cost is difficult in this context |
| **14** | **Govil and Hao, 2016** | Theoretical | Role of clinician in early device development | Basic research on mechanisms and targeting for specific product | Clinicians can help by:   1. Identifying gaps in knowledge 2. Identify clinical needs 3. Develop iterative clinical trials that will eventually address regulatory requirements 4. Engage with specialty medical associations early | - Early engagement with regulatory societies will allow for feedback on the area of clinical need, potential for implementation of device into guidelines and their stand on supporting recommendations for reimbursement |
| **15** | **Hamilton 2007** | Theoretical | Role of clinician in early device development | Product and prototype development | Clinicians can help by:   1. Assessing device efficacy and safety 2. Have appropriate surveillance systems to feedback adverse events associated with device use (i.e to manufacturers and ethics committees) 3. Early involvement of ethics committee to provide support to device investigation efforts 4. Being proactive in being device experts through extensive literature review | - The literature review can focus on the associated clinical problem, precedent device use and issues encountered. - Literature can inform standard of care, primary outcomes for effectiveness testing and potential restrictions |
| **16** | **Hulstaert et al , 2012** | Theoretical | Role of the clinician in early **high risk device** development | Product and prototype development and feasibility studies | Clinicians can help by:   1. Studying device efficacy 2. Studying device safety to reduce patient exposure to a high risk procedure 3. Assessing handling of device and methods to reduce learning curve 4. Designing appropriate studies for the various regulatory pathways 5. Being aware of incremental innovation and its impact on study timing | - For Europe, a demonstration of device safety and performance for intended use as per the manufacturer - For FDA there is a necessity to demonstrate device safety and effectiveness – high dependency of RCT as a result |
| **17** | **Hummel J et al, 2000** | Application | Role of the clinician in early health technology assessment | All phases of EHTA | Proposing innovation model termed constructive MTA. Assesses technologies in terms of clinical , technical, economic and social factors  Method involves comparison of novel technology with available comparable device on factors such as device performance, safety , ease of use and applicability | - Utilises an analytical hierarchy model to allow for quantitative analysis of diverse factors and stakeholders in the medical device process - Can be utilised to provide a broad analysis ,quantify and stratify the importance of design input factors in the early innovation process |
| **18** | **Huygens et al, 2016** | Theoretical | Methods of developing a conceptual model for EHTA | Basic research on mechanisms and targeting for specific product | In building a conceptual model for an area of clinical need, the steps include:   1. Literature review 2. Expert opinion 3. Available cost effectiveness analyses in the area of interest | - Literature review allows for clinicians to outline the clinical problem, population of interest, available alternatives at present, stakeholder perspectives and significant outcomes - Experts allow for feedback regarding the design of models, the interactions of their factors and their relevance to actual clinical practice |
| 19 | **Kesselheim et al. , 2014** | Theoretical Case study – Coronory stent | Factors contributing to successful early device innovation | All phases of EHTA | Factors associated with successful innovation included:   1. Direct exposure to the clinical problem for the clinicians 2. Individual ability to identify innovative solutions to the problem 3. Resilience to adversity in early innovation   Collaborative work between clinicians and other stakeholders in the medical device ecosystem |  |
| **20** | **Lu , 2014** | Theoretical | Approaches to generating better clinical evidence for early stage devices | Prototype and product development | Better clinical evidence can be generated by:   1. Comparative effectiveness research (CER)   Pragmatic clinical trials (PCTS) | - CER mandates head to head comparison of active treatments, designing studies that have external validity and focussing on patient preferences which may inform treatment choices and outcomes   PCRs mandates comparison between devices and clinically relevant treatment alternatives and focuses on measuring clinically relevant outcomes |
| **21** | **Maetzler et al , 2016** | Theoretical  Case study- Sensors in parkinson’s disease | Role of clinician in early device development | Basic research on mechanisms and targeting for specific product | Clinicians can help evaluate the following through feasibility studies :   1. Technical validation of early devices 2. Measuring clinical effectiveness of devices 3. Side effect profile   Impact on QOL |  |
| **22** | **Markiewicz K et al , 2014** | Application | Identification of aims and methods used in EHTA | All phases of EHTA | The primary focus of EHTA include:   1. Strategic considerations 2. Economic evaluation 3. Clinical considerations | - Strategic considerations can be elicited by literature review and early stakeholder involvement - Clinical assessment can begin and influence EHTA from early in the innovation process. Methods are sparse. |
| **23** | **Migliore et al , 2009** | Theoretical | Generation of information regarding area of clinical need | Basic research on mechanisms and targeting for specific product | The nature of the clinical issue can be scoped by :   1. Expert clinician opinion on the issue 2. Systematic review on the issue 3. Information provided by the manufacturer on the device 4. National health registries to obtain information | - Manufacturers seldom have strong evidence to back due to it being a lower priority - SR are hard to derive data from due to the lack of high quality studies to inform them |
| **24** | **Mike V et al** | Theoretical Case study- Saturation monitors for newborns | Industry perspective on needs analysis | Basic research on mechanisms and targeting for specific product | Industry gain insight for needs based development based on:   1. Literature defining a clinical gap 2. Interaction with leading experts in the field 3. Utilising focus groups with clinicians 4. Hiring own consultants | - Companies found that clinicians often had a poor physiological understanding of the problem area - Companies noted that clinicians played a huge role in adoption or abandonment of technology |
| **25** | **Pietzch & Pate Cornell, 2006** | Application | Modelling to inform stakeholders during EHTA | All areas of EHTA | Early modelling in EHTA allows for :   1. Assessment of the likely, safety, effectiveness and cost effectiveness 2. Decision support for manufacturers and investors   Utilises evidence from early clinical evidence or predicates devices | - Focus of study is on using Bayesian methods to assess performance parameters - Modelling allows for main outcomes to be discerned early and aids in risk reduction - Early data can be derived from early clinical use of the device or similar devices, published data , bench data or expert opinion |
| **26** | **Raab & Parr, 2006** | Theoretical | Awareness of reimbursement decisions in early device innovation | Basic research on mechanisms | Reimbursement decisions :   1. Can be predicted when the innovation is incremental in nature 2. Difficult to predict when device is a breakthrough innovation 3. Are affected by learning curves of early use ( device ‘effectiveness’ may differ with ongoing use), patient populations which it may benefit ( could change during the innovation process) | - Even when incremental, the use case of the device may have changed by the time it is available in the market - Reimbursement may be affected by off label use of the device - Breakthrough innovations are hard to predict as its usage depends on effectiveness and physician utilisation post market access |
| **27** | **Ray et al ., 2017** | Theoretical | Needs based innovation approach to device innovation | Basic research on mechanisms and targeting for specific product | Needs based innovation focuses on:   1. Strong clinical needs to justify device development 2. Through understanding of the disease process and areas of care in which the device can affect outcomes 3. Disease and specialty based focuses for device design for deeper understanding | - The needs based innovation is touched upon heavily by the Stanford Biodesign Process - Main focus of needs based innovation is to reduce disease burden, increase access, increase satisfaction and reduce costs - J&J model focuses on the clinicians being in the process of early development – also liaising with professional bodies for clinical guideline development for new therapies |
| **28** | **Schnell- Inderest et al, 2015** | Application | Methodological limitations to assessing medical devices | Proof of principle and prototype and product development | Limitations to assessing MDs include :   1. Incremental innovation associated with it 2. Deciding on the right time to assesses the devices 3. Learning curve associated with its use 4. Methods to group ‘similar’ MDs together and assume thy have the same performance | - Following factors result in low quality evidence being generated for the device - Adaptive trial designs need to be considered to address these issues |
| **29** | **Shah and Robinson ,2006** | Application | Stages of early stage medical device innovation where user perspective can be included | All phases of EHTA | Users can contribute to early innovation process across all phases of EHTA. Highest involvement is during the design phase and testing and trials.  Their role involves assessment and development which feedback into the device process | - Table II in the study provides a comprehensive lists of methods to illicit use perspectives - Financial implications of use involvement needs to be considered |
| **30** | **Shah and Robinson, 2007** | Application | Manners in which user perspective can influence early device innovation | All phase of EHTA | Benefits of user involvement include :   1. User perspectives to aid the innovation process 2. Idea generation 3. Better user and manufacturer interaction 4. Engagement with lead users | - Users aid in generating innovative changes , flag potential pain points and provided early feedback on expectations and product experience |
| 31 | **Shah et al , 2009** | Theoretical | Stages of early stage medical device innovation where user perspective can be included | All phases of EHTA | User feedback can be elicited across:   1. Concept stage 2. Design stage 3. Testing and trials stage   Methods include: Interviews, surveys, focus groups , field observations etc | - Users can be classified as professional and non professional users. Professional users are healthcare professionals and non professional users are the general population using the device with no formal training |
| **32** | **Steinberger et al** | Theoretical | How to identify unmet clinical needs effectively | Basic research on mechanism | Methods to engage in identifying the unmet clinical need include:   1. Assembling a team with a varied skill mix 2. Needs finding and validation 3. Generation of needs statement and criteria 4. Brainstorming for solutions | - Teams that perform well often have a medical, engineering and business expertise - Clinicians plays a big role in needs finding |
| **33** | **Vecht et al** | Theoretical | Identifying mechanisms by which the clinician can influence early device innovation | Basic research mechanisms and prototype and product development | Mechanisms to influence early device innovation include   1. Identifying and characterising the clinical problem 2. Prototype development through feedback | - Feedback to influence prototype development comes from a variety of stakeholders in the process ( i.e engineers, scientists, business) |

**Supplementary Information B : Data extracted from studies to delineate areas in which clinicians can contribute to the EHTA process.**
