# Supplementary material for: Defining the clinician’s role in early health technology assessment during medical device innovation – a systematic review
Source: BMC Health Serv Res. 2019 Jul 23;19:514. doi: 10.1186/s12913-019-4305-9 (PMC6651962; doi:10.1186/s12913-019-4305-9)
Supplement: Supplementary file 3 — NICE qualitative appraisal checklist. (PDF 1073 kb) [file 12913_2019_4305_MOESM3_ESM.pdf]

Checklist

**Study identification:** Include author, title, reference, year of publication		
**Guidance topic:**	**Key research question/aim:**	
**Checklist completed by:**		
**Theoretical approach**		
**1. Is a qualitative approach appropriate?**<br>For example:<br>• Does the research question seek to understand processes or structures, or illuminate subjective experiences or meanings?<br>• Could a quantitative approach better have addressed the research question?	Appropriate<br>Inappropriate<br>Not sure	Comments:
**2. Is the study clear in what it seeks to do?**<br>For example:<br>• Is the purpose of the study discussed – aims/objectives/research question/s?<br>• Is there adequate/appropriate reference to the literature?<br>• Are underpinning values/assumptions/theory discussed?	Clear<br>Unclear<br>Mixed	Comments:
**Study design**		

© NICE 2012. All rights reserved.

**3. How defensible/rigorous is the research design/ methodology?**<br>For example:<br>• Is the design appropriate to the research question?<br>• Is a rationale given for using a qualitative approach?<br>• Are there clear accounts of the rationale/justification for the sampling, data collection and data analysis techniques used?<br>• Is the selection of cases/sampling strategy theoretically justified?	Defensible<br>Indefensible<br>Not sure	Comments:
Data collection		
**4. How well was the data collection carried out?**<br>For example:<br>• Are the data collection methods clearly described?<br>• Were the appropriate data collected to address the research question?<br>• Was the data collection and record keeping systematic?	Appropriately<br>Inappropriately<br>Not sure/ inadequately reported	Comments:
Trustworthiness		
**5. Is the role of the researcher clearly described?**<br>For example:<br>• Has the relationship between the researcher and the participants been adequately considered?<br>• Does the paper describe how the research was explained and presented to the participants?	Clearly described<br>Unclear<br>Not described	Comments:

© NICE 2012. All rights reserved.

**6. Is the context clearly described?**<br>For example:<br>• Are the characteristics of the participants and settings clearly defined?<br>• Were observations made in a sufficient variety of circumstances<br>• Was context bias considered	Clear<br>Unclear<br>Not sure	Comments:
**7. Were the methods reliable?**<br>For example:<br>• Was data collected by more than 1 method?<br>• Is there justification for triangulation, or for not triangulating?<br>• Do the methods investigate what they claim to?	Reliable<br>Unreliable<br>Not sure	Comments:
Analysis		
**8. Is the data analysis sufficiently rigorous?**<br>For example:<br>• Is the procedure explicit – i.e. is it clear how the data was analysed to arrive at the results?<br>• How systematic is the analysis, is the procedure reliable/dependable?<br>• Is it clear how the themes and concepts were derived from the data?	Rigorous<br>Not rigorous<br>Not sure/not reported	Comments:

© NICE 2012. All rights reserved.

**9. Is the data 'rich'?**<br>For example:<br>• How well are the contexts of the data described?<br>• Has the diversity of perspective and content been explored?<br>• How well has the detail and depth been demonstrated?<br>• Are responses compared and contrasted across groups/sites?	Rich<br>Poor<br>Not sure/not reported	Comments:
**10. Is the analysis reliable?**<br>For example:<br>• Did more than 1 researcher theme and code transcripts/data?<br>• If so, how were differences resolved?<br>• Did participants feed back on the transcripts/data if possible and relevant?<br>• Were negative/discrepant results addressed or ignored?	Reliable<br>Unreliable<br>Not sure/not reported	Comments:
**11. Are the findings convincing?**<br>For example:<br>• Are the findings clearly presented?<br>• Are the findings internally coherent?<br>• Are extracts from the original data included?<br>• Are the data appropriately referenced?<br>• Is the reporting clear and coherent?	Convincing<br>Not convincing<br>Not sure	Comments:
**12. Are the findings relevant to the aims of the study?**	Relevant<br>Irrelevant<br>Partially relevant	Comments:

© NICE 2012. All rights reserved.

**13. Conclusions**<br>For example:<br>• How clear are the links between data, interpretation and conclusions?<br>• Are the conclusions plausible and coherent?<br>• Have alternative explanations been explored and discounted?<br>• Does this enhance understanding of the research topic?<br>• Are the implications of the research clearly defined?<br>Is there adequate discussion of any limitations encountered?	Adequate<br>Inadequate<br>Not sure	Comments:
Ethics		
**14. How clear and coherent is the reporting of ethics?**<br>For example:<br>• Have ethical issues been taken into consideration?<br>• Are they adequately discussed e.g. do they address consent and anonymity?<br>• Have the consequences of the research been considered i.e. raising expectations, changing behaviour?<br>• Was the study approved by an ethics committee?	Appropriate<br>Inappropriate<br>Not sure/not reported	Comments:
**Overall assessment**		
**As far as can be ascertained from the paper, how well was the study conducted? (see guidance notes)**	++<br>+<br>−	Comments:

Notes on the use of the qualitative studies checklist

Section 1: theoretical approach

This section deals with the underlying theory and principles applied to the research.

© NICE 2012. All rights reserved.
